# A comparison of RNA folding measures

**DOI:** 10.1186/1471-2105-6-241

**Published:** 2005-10-03

**Authors:** Eva Freyhult, Paul P Gardner, Vincent Moulton

**Affiliations:** 1 The Linnaeus Centre for Bioinformatics, Uppsala University, Uppsala, Sweden.; 2 Dept. of Evolutionary Biology, University of Copenhagen, Universitetsparken 15, 2100 Copenhagen Ø, Denmark.; 3 School of Computing Sciences, University of East Anglia, Norwich, NR4 7TJ, UK.

## Abstract

**Background:**

In the last few decades there has been a great deal of discussion concerning whether or not noncoding RNA sequences (ncRNAs) fold in a more well-defined manner than random sequences. In this paper, we investigate several existing measures for how well an RNA sequence folds, and compare the behaviour of these measures over a large range of Rfam ncRNA families. Such measures can be useful in, for example, identifying novel ncRNAs, and indicating the presence of alternate RNA foldings.

**Results:**

Our analysis shows that ncRNAs, but not mRNAs, in general have lower minimal free energy (MFE) than random sequences with the same dinucleotide frequency. Moreover, even when the MFE is significant, many ncRNAs appear to not have a unique fold, but rather several alternative folds, at least when folded *in silico*. Furthermore, we find that the six investigated measures are correlated to varying degrees.

**Conclusion:**

Due to the correlations between the different measures we find that it is sufficient to use only two of them in RNA folding studies, one to test if the sequence in question has lower energy than a random sequence with the same dinucleotide frequency (the *Z*-score) and the other to see if the sequence has a unique fold (the average base-pair distance, *D*).

## Background

Noncoding RNAs (ncRNAs) are sequences that are transcribed from DNA that function as RNA rather than being translated to protein. Many of the known ncRNAs, such as transfer RNA (tRNA), ribosomal RNA (rRNA), spliceosomal RNA (snRNA), and microRNAs (miRNA), have key functions in the cell. Moreover, various new families of ncRNAs are emerging, and, as indicated in recent studies in mouse [1] and 10 human chromosomes [2], many more transcripts are for ncRNAs than was previously expected. In the late 1980's Maizel and co-workers proposed the use of thermodynamic stability to identify noncoding RNAs in sequence data [3-5]. Since then, there has been a great deal of discussion concerning whether or not ncRNA sequences support secondary structure features that are significantly different from those of random sequences. In particular, following some contradictory results concerning the stability of messenger RNAs (mRNA) presented in [6-8], in [9] it was concluded that ncRNAs have more stable structures than random sequences, but that the difference is not significant enough to be of use in identifying novel RNAs in sequence data on its own (see also [10]). Even so, more recent findings suggest that thermodynamic stability can be used to identify novel members of special families of RNAs [11], and that stability coupled with comparative genomics data is a useful tool for identifying ncRNAs in general [12].

To shed more light on the above findings, we present a large scale investigation for how well ncRNA sequences fold compared with random sequences. In particular, we investigate six measures for how well an RNA sequence folds (normalised energy (*dG*), *Z*-score (*Z*) and *p*-value (*p*) of minimal free energy (MFE), Shannon entropy (*Q*), average base pair distance (*D*), and valley index (*VI*), for definitions see the Methods section), and compare the behaviour of these measures over a large range of Rfam ncRNA families (see Table 1), including many of the families that appeared in the studies mentioned above.

**Table 1:** The data sets used in this study. The first column contains a short name describing the data set, that is later used in text and figures. The RNA families/data sets can contain several types of sequences, such as the RNase family that contains both RNase P and RNase MRP. The different sequence types, or family members, are given in column two. In column three and four the number of family members (*N*_*FM*_) and the total number of sequences (*N*_*S*_) are given, respectively. The last two columns in the table give the mean and standard deviation of the sequence length and %GC-content.

short name (full name)	family members	*N*_*S*_	length	% GC
miRNA (microRNA)	all 38 miRNAs in Rfam	135	82.82 ± 16.01	46.01 ± 7.44
intron	group I and II	107	148.03 ± 113.72	43.24 ± 10.33
RNase	RNase P and MRP	147	320.89 ± 37.80	56.70 ± 9.88
SRP (signal recognition particle)	bacterial and eukaryotic/archae SRP	77	187.14 ± 100.47	58.27 ± 10.44
rRNA (ribosomal RNA)	small subunit and 5S rRNA	578	380.06 ± 196.50	50.93 ± 8.37
snRNA (small nuclear spliceosomal RNA)	all 8 spliceosomal snRNAs in Rfam	82	135.22 ± 39.40	47.19 ± 6.69
riboswitch	lysine, s-box (SAM riboswitch), cobalamin	154	175.87 ± 49.59	51.38 ± 10.33
tmRNA		59	345.03 ± 32.18	45.48 ± 10.07
regulatory	IRE, IRES, SECIS, HIV primer binding site, VARNA	17	80.62 ± 56.24	49.78 ± 10.21
tRNA (transfer RNA)		565	73.16 ± 5.41	46.94 ± 12.02
telomerase		17	442.53 ± 41.23	64.49 ± 6.98
snoRNA (small nucleolar RNA)	all 177 guide snoRNAs in Rfam	412	97.60 ± 39.64	43.38 ± 7.43
Hh1 (Hammerhead ribozyme (type I))		16	54.44 ± 24.08	49.49 ± 8.03
mRNA (messenger RNA)		32	329.94 ± 90.33	49.98 ± 8.47
shuffled (control data set)		130	199.86 ± 154.01	50.33 ± 10.61

## Methods

### Data sets

All data sets, except the protein control and the ribosomal RNA data sets, were obtained from Rfam 6.1 [13]. Rfam seed alignments were used to select a collection of RNA families, which are specified in Table 1. The rRNA data set consists of a large representative subset of the eukaryotic SSU rRNA sequences in the European rRNA database (see additional file 1  for further details).

For each class of RNA we obtained an alignment of sequences, which we filtered so that it had no more than 80% sequence identity. This was done using the program weight, that is part of the Sean Eddy "squid" utilities (downloaded 2004 from ).

In addition to the 13 data sets specified in Table 1, two control data sets were included; a protein control data set, consisting of 32 small protein coding sequences, and a set of shuffled RNA sequences (see additional file 1 for further details). The shuffled data set consists of 10 sequences from each of the 13 RNA data sets that were permuted, preserving dinucleotide frequencies [14], resulting in 130 sequences.

### RNA folding statistics

Several quantities have been proposed for predicting how well an RNA molecule folds. In this paper we consider the following: The normalised minimal free energy (MFE) per base-pair (*dG*), the *Z*-score (*Z*), the *p*-value (*p*), the Shannon entropy (*Q*), the average base-pair distance (*D*), and the valley index (*VI*). We now present formal definitions for each of these measures. Let **x **= *x*_1 _⋯ *x*_*L*_, denote an RNA sequence of length *L*, so that *x*_*i *_is either A, C, G or U for each 1 ≤ *i *≤ *L*.

The normalised energy, *dG*, is arrived at from a free energy minimisation procedure. It is defined as

dG(x):=E(x)L,
 MathType@MTEF@5@5@+=feaafiart1ev1aaatCvAUfKttLearuWrP9MDH5MBPbIqV92AaeXatLxBI9gBaebbnrfifHhDYfgasaacH8akY=wiFfYdH8Gipec8Eeeu0xXdbba9frFj0=OqFfea0dXdd9vqai=hGuQ8kuc9pgc9s8qqaq=dirpe0xb9q8qiLsFr0=vr0=vr0dc8meaabaqaciaacaGaaeqabaqabeGadaaakeaacqWGKbazcqWGhbWrcqGGOaaktCvAUfeBSjuyZL2yd9gzLbvyNv2CaeHbwvMCKfMBHbaceeGaa8hEaGabaiaa+LcacaGF6aGaa4xpamaalaaabaGaemyrauKaeiikaGIaa8hEaiaa+LcaaeaacqWGmbataaGaeiilaWcaaa@433B@

where *E*(**x**) is the minimal free energy (MFE) for sequence **x**, as computed using RNAfold [15]. This program implements the folding algorithm presented in [16].

The *Z*-score and the *p*-value compare the MFE of the sequence **x **to the MFEs of permuted versions of **x **having identical dinucleotide composition. These compositions are preserved due to the importance of stacked base-pairs in the calculation of MFE [8]. For each sequence in this study, 500 shuffled sequences were generated using a mono- and dinucleotide frequency preserving procedure implemented in the program shuffle that is part of the Sean Eddy "squid" utilities.

The *Z*-score [17] is the number of standard deviations by which the MFE of **x **deviates from the mean MFE of the set Xshuffled(x)
 MathType@MTEF@5@5@+=feaafiart1ev1aaatCvAUfKttLearuWrP9MDH5MBPbIqV92AaeXatLxBI9gBamXvP5wqSXMqHnxAJn0BKvguHDwzZbqegm0B1jxALjhiov2DaebbnrfifHhDYfgasaacH8akY=wiFfYdH8Gipec8Eeeu0xXdbba9frFj0=OqFfea0dXdd9vqai=hGuQ8kuc9pgc9s8qqaq=dirpe0xb9q8qiLsFr0=vr0=vr0dc8meaabaqaciaacaGaaeqabaWaaeGaeaaakeaaimaacaWFybWaaSbaaSqaaeHbnf2C0vMCJfMCKbacfiGaa43Caiaa+HgacaGF1bGaa4Nzaiaa+zgacaGFSbGaa4xzaiaa+rgaaeqaaOGaeiikaGsegyvzYrwyUfgaiyqacaqF4bGaeiykaKcaaa@4AB6@ of shuffled sequences [6,8,9,17]. It is defined as

Z(x):=E(x)−<Xshuffled(x)>σ(Xshuffled(x)),
 MathType@MTEF@5@5@+=feaafiart1ev1aaatCvAUfKttLearuWrP9MDH5MBPbIqV92AaeXatLxBI9gBamXvP5wqSXMqHnxAJn0BKvguHDwzZbqegm0B1jxALjhiov2DaebbnrfifHhDYfgasaacH8akY=wiFfYdH8Gipec8Eeeu0xXdbba9frFj0=OqFfea0dXdd9vqai=hGuQ8kuc9pgc9s8qqaq=dirpe0xb9q8qiLsFr0=vr0=vr0dc8meaabaqaciaacaGaaeqabaWaaeGaeaaakeaacqWGAbGwcqGGOaakryGvLjhzH5wyaGqbbiaa=HhacqGGPaqkcqGG6aGocqGH9aqpdaWcaaqaaiabdweafjabcIcaOiaa=HhacqGGPaqkcqGHsislcqGH8aapimaacaGFybWaaSbaaSqaaeHbnf2C0vMCJfMCKbacgiGaa03Caiaa9HgacaqF1bGaa0Nzaiaa9zgacaqFSbGaa0xzaiaa9rgaaeqaaOGaeiikaGIaa8hEaiabcMcaPiabg6da+aqaaGGaciab8n8aZjabcIcaOiaa+HfadaWgaaWcbaGaa03Caiaa9HgacaqF1bGaa0Nzaiaa9zgacaqFSbGaa0xzaiaa9rgaaeqaaOGaeiikaGIaa8hEaiabcMcaPiabcMcaPaaacqGGSaalaaa@66BB@

where <·> and *σ*(·) denote the mean and the standard deviation of the MFEs of the sequences in Xshuffled(x)
 MathType@MTEF@5@5@+=feaafiart1ev1aaatCvAUfKttLearuWrP9MDH5MBPbIqV92AaeXatLxBI9gBamXvP5wqSXMqHnxAJn0BKvguHDwzZbqegm0B1jxALjhiov2DaebbnrfifHhDYfgasaacH8akY=wiFfYdH8Gipec8Eeeu0xXdbba9frFj0=OqFfea0dXdd9vqai=hGuQ8kuc9pgc9s8qqaq=dirpe0xb9q8qiLsFr0=vr0=vr0dc8meaabaqaciaacaGaaeqabaWaaeGaeaaakeaaimaacaWFybWaaSbaaSqaaeHbnf2C0vMCJfMCKbacfiGaa43Caiaa+HgacaGF1bGaa4Nzaiaa+zgacaGFSbGaa4xzaiaa+rgaaeqaaOGaeiikaGsegyvzYrwyUfgaiyqacaqF4bGaeiykaKcaaa@4AB6@.

The *p*-value of **x **is the fraction of sequences in Xshuffled(x)
 MathType@MTEF@5@5@+=feaafiart1ev1aaatCvAUfKttLearuWrP9MDH5MBPbIqV92AaeXatLxBI9gBamXvP5wqSXMqHnxAJn0BKvguHDwzZbqegm0B1jxALjhiov2DaebbnrfifHhDYfgasaacH8akY=wiFfYdH8Gipec8Eeeu0xXdbba9frFj0=OqFfea0dXdd9vqai=hGuQ8kuc9pgc9s8qqaq=dirpe0xb9q8qiLsFr0=vr0=vr0dc8meaabaqaciaacaGaaeqabaWaaeGaeaaakeaaimaacaWFybWaaSbaaSqaaeHbnf2C0vMCJfMCKbacfiGaa43Caiaa+HgacaGF1bGaa4Nzaiaa+zgacaGFSbGaa4xzaiaa+rgaaeqaaOGaeiikaGsegyvzYrwyUfgaiyqacaqF4bGaeiykaKcaaa@4AB6@ having MFE lower than **x **or, expressed differently, the area under the distribution function to the left of the MFE of **x**. It is defined as

p(x):=MN,
 MathType@MTEF@5@5@+=feaafiart1ev1aaatCvAUfKttLearuWrP9MDH5MBPbIqV92AaeXatLxBI9gBaebbnrfifHhDYfgasaacH8akY=wiFfYdH8Gipec8Eeeu0xXdbba9frFj0=OqFfea0dXdd9vqai=hGuQ8kuc9pgc9s8qqaq=dirpe0xb9q8qiLsFr0=vr0=vr0dc8meaabaqaciaacaGaaeqabaqabeGadaaakeaacqWGWbaCcqGGOaaktCvAUfeBSjuyZL2yd9gzLbvyNv2CaeHbwvMCKfMBHbaceeGaa8hEaGabaiaa+LcacqGG6aGocqGH9aqpdaWcaaqaaiabd2eanbqaaiabd6eaobaacqGGSaalaaa@4061@

where *M *is the number of sequences in Xshuffled(x)
 MathType@MTEF@5@5@+=feaafiart1ev1aaatCvAUfKttLearuWrP9MDH5MBPbIqV92AaeXatLxBI9gBamXvP5wqSXMqHnxAJn0BKvguHDwzZbqegm0B1jxALjhiov2DaebbnrfifHhDYfgasaacH8akY=wiFfYdH8Gipec8Eeeu0xXdbba9frFj0=OqFfea0dXdd9vqai=hGuQ8kuc9pgc9s8qqaq=dirpe0xb9q8qiLsFr0=vr0=vr0dc8meaabaqaciaacaGaaeqabaWaaeGaeaaakeaaimaacaWFybWaaSbaaSqaaeHbnf2C0vMCJfMCKbacfiGaa43Caiaa+HgacaGF1bGaa4Nzaiaa+zgacaGFSbGaa4xzaiaa+rgaaeqaaOGaeiikaGsegyvzYrwyUfgaiyqacaqF4bGaeiykaKcaaa@4AB6@ with MFE lower than the MFE of **x**, and *N *is the number of shuffled sequences, |Xshuffled(x)
 MathType@MTEF@5@5@+=feaafiart1ev1aaatCvAUfKttLearuWrP9MDH5MBPbIqV92AaeXatLxBI9gBamXvP5wqSXMqHnxAJn0BKvguHDwzZbqegm0B1jxALjhiov2DaebbnrfifHhDYfgasaacH8akY=wiFfYdH8Gipec8Eeeu0xXdbba9frFj0=OqFfea0dXdd9vqai=hGuQ8kuc9pgc9s8qqaq=dirpe0xb9q8qiLsFr0=vr0=vr0dc8meaabaqaciaacaGaaeqabaWaaeGaeaaakeaaimaacaWFybWaaSbaaSqaaeHbnf2C0vMCJfMCKbacfiGaa43Caiaa+HgacaGF1bGaa4Nzaiaa+zgacaGFSbGaa4xzaiaa+rgaaeqaaOGaeiikaGsegyvzYrwyUfgaiyqacaqF4bGaeiykaKcaaa@4AB6@|.

*In vivo*, RNAs commonly exist in an ensemble of structures. The distribution of these structures can be modelled by a Boltzmann distribution. Using this setup, it is possible to efficiently compute the partition function, *Z*, for the ensemble S(x)
 MathType@MTEF@5@5@+=feaafiart1ev1aaatCvAUfKttLearuWrP9MDH5MBPbIqV92AaeXatLxBI9gBaebbnrfifHhDYfgasaacH8akY=wiFfYdH8Gipec8Eeeu0xXdbba9frFj0=OqFfea0dXdd9vqai=hGuQ8kuc9pgc9s8qqaq=dirpe0xb9q8qiLsFr0=vr0=vr0dc8meaabaqaciaacaGaaeqabaqabeGadaaakeaatCvAUfeBSjuyZL2yd9gzLbvyNv2CaeHbbjxAHXgaiqaacaWFtbGaeiikaGsegyvzYrwyUfgaiuqacaGF4bGaeiykaKcaaa@3CC3@ of secondary structures corresponding to an RNA sequence **x **[18]. In particular, the probability of a structure *S*_*α *_∊ S(x)
 MathType@MTEF@5@5@+=feaafiart1ev1aaatCvAUfKttLearuWrP9MDH5MBPbIqV92AaeXatLxBI9gBaebbnrfifHhDYfgasaacH8akY=wiFfYdH8Gipec8Eeeu0xXdbba9frFj0=OqFfea0dXdd9vqai=hGuQ8kuc9pgc9s8qqaq=dirpe0xb9q8qiLsFr0=vr0=vr0dc8meaabaqaciaacaGaaeqabaqabeGadaaakeaatCvAUfeBSjuyZL2yd9gzLbvyNv2CaeHbbjxAHXgaiqaacaWFtbGaeiikaGsegyvzYrwyUfgaiuqacaGF4bGaeiykaKcaaa@3CC3@ (which we regard as a set of base-pairs) is given by P(Sα)=e−Eα/RTZ
 MathType@MTEF@5@5@+=feaafiart1ev1aaatCvAUfKttLearuWrP9MDH5MBPbIqV92AaeXatLxBI9gBaebbnrfifHhDYfgasaacH8akY=wiFfYdH8Gipec8Eeeu0xXdbba9frFj0=OqFfea0dXdd9vqai=hGuQ8kuc9pgc9s8qqaq=dirpe0xb9q8qiLsFr0=vr0=vr0dc8meaabaqaciaacaGaaeqabaqabeGadaaakeaacqWGqbaucqGGOaakcqWGtbWudaWgaaWcbaacciGae8xSdegabeaakiabcMcaPiabg2da9maalaaabaGaemyzau2aaWbaaSqabeaacqGHsislcqWGfbqrdaWgaaadbaGae8xSdegabeaaliabc+caViabdkfasjabdsfaubaaaOqaaiabdQfaAbaaaaa@3D85@, where Z=∑Sα∈S(x)e−Eα/RT
 MathType@MTEF@5@5@+=feaafiart1ev1aaatCvAUfKttLearuWrP9MDH5MBPbIqV92AaeXatLxBI9gBamrtHrhAL1wy0L2yHvtyaeHbnfgDOvwBHrxAJfwnaebbnrfifHhDYfgasaacH8akY=wiFfYdH8Gipec8Eeeu0xXdbba9frFj0=OqFfea0dXdd9vqai=hGuQ8kuc9pgc9s8qqaq=dirpe0xb9q8qiLsFr0=vr0=vr0dc8meaabaqaciaacaGaaeqabaWaaeGaeaaakeaacqWGAbGwcqGH9aqpdaaeqaqaaiabdwgaLnaaCaaaleqabaGaeyOeI0Iaemyrau0aaSbaaWqaaGGaciab=f7aHbqabaWccqGGVaWlcqWGsbGucqWGubavaaaabaGaem4uam1aaSbaaWqaaiab=f7aHbqabaWccqGHiiIZt0uy0HwzTfgDPnwy3aqeh0uy0HwzTfgDPnwy3aacfaGae4NKWpLaeiikaGYexLMBbXgBcf2CPn2qVrwzqf2zLnharGGvLjhzH5wyaGGbbiaa9HhacqGGPaqkaeqaniabggHiLdaaaa@6095@ , *E*_*α *_is the free energy of *S*_*α*_, *R *= 8.31451 Jmol^-1^K^-1 ^is the molar gas constant, and *T *is the temperature, which we take as 310.15 K (37°C). The base-pair probability *p*_*ij *_(the probability that *x*_*i *_pairs with *x*_*j*_) is then given by pij=∑Sα∈S(x)P(Sα)δijα
 MathType@MTEF@5@5@+=feaafiart1ev1aaatCvAUfKttLearuWrP9MDH5MBPbIqV92AaeXatLxBI9gBamrtHrhAL1wy0L2yHvtyaeHbnfgDOvwBHrxAJfwnaebbnrfifHhDYfgasaacH8akY=wiFfYdH8Gipec8Eeeu0xXdbba9frFj0=OqFfea0dXdd9vqai=hGuQ8kuc9pgc9s8qqaq=dirpe0xb9q8qiLsFr0=vr0=vr0dc8meaabaqaciaacaGaaeqabaWaaeGaeaaakeaacqWGWbaCdaWgaaWcbaGaemyAaKMaemOAaOgabeaakiabg2da9maaqababaGaemiuaaLaeiikaGIaem4uam1aaSbaaSqaaGGaciab=f7aHbqabaGccqGGPaqkcqWF0oazdaqhaaWcbaGaemyAaKMaemOAaOgabaGae8xSdegaaaqaaiabdofatnaaBaaameaacqWFXoqyaeqaaSGaeyicI48enfgDOvwBHrxAJf2naeXbnfgDOvwBHrxAJf2naGqbaiab+jj8tjab+bW9OiabcIcaOmXvP5wqSXMqHnxAJn0BKvguHDwzZbqeiyvzYrwyUfgaiyqacaqF4bGaeiykaKcabeqdcqGHris5aaaa@68B5@ is 1 if *x*_*i *_and *x*_*j *_is a base-pair in *S*_*α*_, and 0 otherwise.

We use the implementation of McCaskill's algorithm in RNAfold to compute base-pair probabilities. The normalised Shannon entropy of **x **[19] is then defined as

Q(x):=−∑i<jpijlog⁡2(pij)L.
 MathType@MTEF@5@5@+=feaafiart1ev1aaatCvAUfKttLearuWrP9MDH5MBPbIqV92AaeXatLxBI9gBaebbnrfifHhDYfgasaacH8akY=wiFfYdH8Gipec8Eeeu0xXdbba9frFj0=OqFfea0dXdd9vqai=hGuQ8kuc9pgc9s8qqaq=dirpe0xb9q8qiLsFr0=vr0=vr0dc8meaabaqaciaacaGaaeqabaqabeGadaaakeaacqWGrbqucqGGOaaktCvAUfeBSjuyZL2yd9gzLbvyNv2CaeHbwvMCKfMBHbaceeGaa8hEaGabaiaa+LcacqGG6aGocqGH9aqpdaWcaaqaaiabgkHiTmaaqababaGaemiCaa3aaSbaaSqaaiabdMgaPjabdQgaQbqabaGccyGGSbaBcqGGVbWBcqGGNbWzdaWgaaWcbaGaeGOmaidabeaakiabcIcaOiabdchaWnaaBaaaleaacqWGPbqAcqWGQbGAaeqaaOGaeiykaKcaleaacqWGPbqAcqGH8aapcqWGQbGAaeqaniabggHiLdaakeaacqWGmbataaGaeiOla4caaa@553C@

We can also use base-pair probabilities to compute the average base pair distance between all structures in S(x)
 MathType@MTEF@5@5@+=feaafiart1ev1aaatCvAUfKttLearuWrP9MDH5MBPbIqV92AaeXatLxBI9gBaebbnrfifHhDYfgasaacH8akY=wiFfYdH8Gipec8Eeeu0xXdbba9frFj0=OqFfea0dXdd9vqai=hGuQ8kuc9pgc9s8qqaq=dirpe0xb9q8qiLsFr0=vr0=vr0dc8meaabaqaciaacaGaaeqabaqabeGadaaakeaatCvAUfeBSjuyZL2yd9gzLbvyNv2CaeHbbjxAHXgaiqaacaWFtbGaeiikaGsegyvzYrwyUfgaiuqacaGF4bGaeiykaKcaaa@3CC3@, <*d*_*BP*_*> *as follows (I.Hofacker, pers. commun.). (Version 1.5beta of RNAfold output this measure as "ensemble diversity".) The base-pair distance, *d*_*BP*_(*S*_*α*_, *S*_*β*_) between two structures *S*_*α *_and *S*_*β *_on **x **is defined as the number of base-pairs not shared by the structures *S*_*α *_and *S*_*β *_(see e.g. [20]). Hence, if |*S*_*α*_| is the number of base-pairs in *S*_*α*_, i.e. |Sα|= ∑i<jδijα'
 MathType@MTEF@5@5@+=feaafiart1ev1aaatCvAUfKttLearuWrP9MDH5MBPbIqV92AaeXatLxBI9gBaebbnrfifHhDYfgasaacH8akY=wiFfYdH8Gipec8Eeeu0xXdbba9frFj0=OqFfea0dXdd9vqai=hGuQ8kuc9pgc9s8qqaq=dirpe0xb9q8qiLsFr0=vr0=vr0dc8meaabaqaciaacaGaaeqabaqabeGadaaakeaacqGG8baFcqWGtbWudaWgaaWcbaacciGae8xSdegabeaakiabcYha8jabb2da9iaaykW7daaeqaqaaiab=r7aKnaaDaaaleaatCvAUfeBSjuyZL2yd9gzLbvyNv2CaeHbwvMCKfMBHbaceiGaa4xAaiaa+PgaaeaacqWFXoqycaGFNaaaaaqaaiabdMgaPjabgYda8iabdQgaQbqab0GaeyyeIuoaaaa@4B45@ , where *i *and *j *lie between 1 and *L*, then the base-pair distance between structures *S*_*α *_and *S*_*β *_equals

dBP(Sα,Sβ)=  |Sα∪Sβ|−|Sα∩Sβ|=|Sα|+|Sβ|−2|Sα∩Sβ|=∑i<j(δijα+  δij−2δijδij).
 MathType@MTEF@5@5@+=feaafiart1ev1aaatCvAUfKttLearuWrP9MDH5MBPbIqV92AaeXatLxBI9gBaebbnrfifHhDYfgasaacH8akY=wiFfYdH8Gipec8Eeeu0xXdbba9frFj0=OqFfea0dXdd9vqai=hGuQ8kuc9pgc9s8qqaq=dirpe0xb9q8qiLsFr0=vr0=vr0dc8meaabaqaciaacaGaaeqabaqabeGadaaakqaaeeqaaiabdsgaKnaaBaaaleaacqWGcbGqcqWGqbauaeqaaOGaeiikaGIaem4uam1aaSbaaSqaaGGaciab=f7aHbqabaGccqGGSaalcqWGtbWudaWgaaWcbaGae8NSdigabeaakiabcMcaPiabg2da9iaaykW7caaMc8UaeiiFaWNaem4uam1aaSbaaSqaaiab=f7aHbqabaGccqWIQisvcqWGtbWudaWgaaWcbaGae8NSdigabeaakiabcYha8jabgkHiTiabcYha8jabdofatnaaBaaaleaacqWFXoqyaeqaaOGaeSykIKKaem4uam1aaSbaaSqaaiab=j7aIbqabaGccqGG8baFcqqG9aqpcqqG8baFcqWGtbWudaWgaaWcbaGae8xSdegabeaakiabcYha8jabgUcaRiabcYha8jabdofatnaaBaaaleaacqWFYoGyaeqaaOGaeiiFaWNaeyOeI0IaeGOmaiJaeiiFaWNaem4uam1aaSbaaSqaaiab=f7aHbqabaGccqWIPisscqWGtbWudaWgaaWcbaGae8NSdigabeaakiabcYha8bqaaiabg2da9maaqafabaGaeiikaGIae8hTdq2aa0baaSqaaiabdMgaPjabdQgaQbqaaiab=f7aHbaakiabgUcaRaWcbaGaemyAaKMaeyipaWJaemOAaOgabeqdcqGHris5aOGaaGPaVlaaykW7cqWF0oazdaqhaaWcbaGaemyAaKMaemOAaOgabaWaaSbaaWqaaiab=j7aIbqabaaaaOGaeyOeI0IaeGOmaiJae8hTdq2aa0baaSqaaiabdMgaPjabdQgaQbqaamaaBaaameaacqWFXoqyaeqaaaaakiab=r7aKnaaDaaaleaacqWGPbqAcqWGQbGAaeaadaWgaaadbaGae8NSdigabeaaaaGccqGGPaqkcqGGUaGlaaaa@94E2@

In particular,

<dBP>=12∑Sα,Sβ∈S(x)[P(Sα)P(Sβ)∑i<j(δijα+δijβ−2δijαδijβ)].
 MathType@MTEF@5@5@+=feaafiart1ev1aaatCvAUfKttLearuWrP9MDH5MBPbIqV92AaeXatLxBI9gBaebbnrfifHhDYfgasaacH8akY=wiFfYdH8Gipec8Eeeu0xXdbba9frFj0=OqFfea0dXdd9vqai=hGuQ8kuc9pgc9s8qqaq=dirpe0xb9q8qiLsFr0=vr0=vr0dc8meaabaqaciaacaGaaeqabaqabeGadaaakeaacqGH8aapcqWGKbazdaWgaaWcbaGaemOqaiKaemiuaafabeaakiabg6da+iabg2da9maalaaabaGaeGymaedabaGaeGOmaidaamaaqafabaGaei4waSLaemiuaaLaeiikaGIaem4uam1aaSbaaSqaaGGaciab=f7aHbqabaGccqGGPaqkcqWGqbaucqGGOaakcqWGtbWudaWgaaWcbaGae8NSdigabeaakiabcMcaPmaaqafabaGaeiikaGIae8hTdq2aa0baaSqaaiabdMgaPjabdQgaQbqaaiab=f7aHbaakiabgUcaRiab=r7aKnaaDaaaleaacqWGPbqAcqWGQbGAaeaacqWFYoGyaaGccqGHsislcqaIYaGmcqWF0oazdaqhaaWcbaGaemyAaKMaemOAaOgabaGae8xSdegaaOGae8hTdq2aa0baaSqaaiabdMgaPjabdQgaQbqaaiab=j7aIbaaaeaacqWGPbqAcqGH8aapcqWGQbGAaeqaniabggHiLdaaleaacqWGtbWudaWgaaadbaGae8xSdegabeaaliabcYcaSiabdofatnaaBaaameaacqWFYoGyaeqaaSGaeyicI48exLMBbXgBcf2CPn2qVrwzqf2zLnharyqqYLwySbaceaGaa43uaiabcIcaOeHbwvMCKfMBHbacfeGaa0hEaiabcMcaPaqab0GaeyyeIuoakiabcMcaPiabc2faDjabc6caUaaa@8222@

Since |Sα|= ∑i<jδijα'
 MathType@MTEF@5@5@+=feaafiart1ev1aaatCvAUfKttLearuWrP9MDH5MBPbIqV92AaeXatLxBI9gBaebbnrfifHhDYfgasaacH8akY=wiFfYdH8Gipec8Eeeu0xXdbba9frFj0=OqFfea0dXdd9vqai=hGuQ8kuc9pgc9s8qqaq=dirpe0xb9q8qiLsFr0=vr0=vr0dc8meaabaqaciaacaGaaeqabaqabeGadaaakeaacqGG8baFcqWGtbWudaWgaaWcbaacciGae8xSdegabeaakiabcYha8jabb2da9iaaykW7daaeqaqaaiab=r7aKnaaDaaaleaatCvAUfeBSjuyZL2yd9gzLbvyNv2CaeHbwvMCKfMBHbaceiGaa4xAaiaa+PgaaeaacqWFXoqycaGFNaaaaaqaaiabdMgaPjabgYda8iabdQgaQbqab0GaeyyeIuoaaaa@4B45@ , <*d*_*BP*_*> *can thus be rewritten as

<dBP>  =  12∑Sα,Sβ∈S(x)[P(Sα)P(Sβ)∑i<j(δijα+δijβ−2δijαδijβ)]                   =12∑i<j[∑Sα∈S(x)P(Sα)δijα︸pij∑Sβ∈S(x)P(Sβ)︸1+∑Sα∈S(x)P(Sα)︸1∑Sβ∈S(x)P(Sβ)δijβ︸pij                        −2∑Sα∈S(x)P(Sα)δijα︸pij∑Sβ∈S(x)P(Sβ)δijβ]︸pij                   =12∑i<j[pij+pij+2pijpij]                   =∑i<j(pij−pij2).
 MathType@MTEF@5@5@+=feaafiart1ev1aaatCvAUfKttLearuWrP9MDH5MBPbIqV92AaeXatLxBI9gBamrtHrhAL1wy0L2yHvtyaeHbnfgDOvwBHrxAJfwnaebbnrfifHhDYfgasaacH8akY=wiFfYdH8Gipec8Eeeu0xXdbba9frFj0=OqFfea0dXdd9vqai=hGuQ8kuc9pgc9s8qqaq=dirpe0xb9q8qiLsFr0=vr0=vr0dc8meaabaqaciaacaGaaeqabaWaaeGaeaaakqaabeqaaiabgYda8iabdsgaKnaaBaaaleaacqWGcbGqcqWGqbauaeqaaOGaeyOpa4JaaGPaVlaaykW7cqGH9aqpcaaMc8UaaGPaVpaalaaabaGaeGymaedabaGaeGOmaidaamaaqafabaGaei4waSLaemiuaaLaeiikaGIaem4uam1aaSbaaSqaaGGaciab=f7aHbqabaGccqGGPaqkcqWGqbaucqGGOaakcqWGtbWudaWgaaWcbaGae8NSdigabeaakiabcMcaPmaaqafabaGaeiikaGIae8hTdq2aa0baaSqaaiabdMgaPjabdQgaQbqaaiab=f7aHbaakiabgUcaRiab=r7aKnaaDaaaleaacqWGPbqAcqWGQbGAaeaacqWFYoGyaaGccqGHsislcqaIYaGmcqWF0oazdaqhaaWcbaGaemyAaKMaemOAaOgabaGae8xSdegaaOGae8hTdq2aa0baaSqaaiabdMgaPjabdQgaQbqaaiab=j7aIbaakiabcMcaPiabc2faDbWcbaGaemyAaKMaeyipaWJaemOAaOgabeqdcqGHris5aaWcbaGaem4uam1aaSbaaWqaaiab=f7aHbqabaWccqGGSaalcqWGtbWudaWgaaadbaGae8NSdigabeaaliabgIGioprtHrhAL1wy0L2yHDdarCqtHrhAL1wy0L2yHDdaiuaacqGFsc=ucqGGOaaktCvAUfeBSjuyZL2yd9gzLbvyNv2CaebcwvMCKfMBHbacgeGaa0hEaiabcMcaPaqab0GaeyyeIuoaaOqaaiaaykW7caaMc8UaaGPaVlaaykW7caaMc8UaaGPaVlaaykW7caaMc8UaaGPaVlaaykW7caaMc8UaaGPaVlaaykW7caaMc8UaaGPaVlaaykW7caaMc8UaaGPaVlaaykW7cqGH9aqpdaWcaaqaaiabigdaXaqaaiabikdaYaaadaaeqbqaaiabcUfaBnaayaaabaWaaabuaeaacqWGqbaucqGGOaakcqWGtbWudaWgaaWcbaGae8xSdegabeaakiabcMcaPiab=r7aKnaaDaaaleaacqWGPbqAcqWGQbGAaeaacqWFXoqyaaaabaGaem4uam1aaSbaaWqaaiab=f7aHbqabaWccqGHiiIZcqGFsc=ucqGGOaakcaqF4bGaeiykaKcabeqdcqGHris5aaWcbaGaemiCaa3aaSbaaWqaaiabdMgaPjabdQgaQbqabaaakiaawIJ=aaWcbaGaemyAaKMaeyipaWJaemOAaOgabeqdcqGHris5aOWaaGbaaeaadaaeqbqaaiabdcfaqjabcIcaOiabdofatnaaBaaaleaacqWFYoGyaeqaaOGaeiykaKcaleaacqWGtbWudaWgaaadbaGae8NSdigabeaaliabgIGiolab+jj8tjabcIcaOiaa9HhacqGGPaqkaeqaniabggHiLdaaleaaiyaacaaFXaaakiaawIJ=aiabgUcaRmaayaaabaWaaabuaeaacqWGqbaucqGGOaakcqWGtbWudaWgaaWcbaGae8xSdegabeaakiabcMcaPaWcbaGaem4uam1aaSbaaWqaaiab=f7aHbqabaWccqGHiiIZcqGFsc=ucqGFaCpkcqGGOaakcaqF4bGaeiykaKcabeqdcqGHris5aaWcbaGaeGymaedakiaawIJ=amaayaaabaWaaabuaeaacqWGqbaucqGGOaakcqWGtbWudaWgaaWcbaGae8NSdigabeaakiabcMcaPiab=r7aKnaaDaaaleaacqWGPbqAcqWGQbGAaeaacqWFYoGyaaaabaGaem4uam1aaSbaaWqaaiab=j7aIbqabaWccqGHiiIZcqGFsc=ucqGGOaakcaqF4bGaeiykaKcabeqdcqGHris5aaWcbaGaemiCaa3aaSbaaWqaaiabdMgaPjabdQgaQbqabaaakiaawIJ=aaqaaiaaykW7caaMc8UaaGPaVlaaykW7caaMc8UaaGPaVlaaykW7caaMc8UaaGPaVlaaykW7caaMc8UaaGPaVlaaykW7caaMc8UaaGPaVlaaykW7caaMc8UaaGPaVlaaykW7caaMc8UaaGPaVlaaykW7caaMc8UaaGPaVlabgkHiTiabikdaYmaayaaabaWaaabuaeaacqWGqbaucqGGOaakcqWGtbWudaWgaaWcbaGae8xSdegabeaakiabcMcaPiab=r7aKnaaDaaaleaacqWGPbqAcqWGQbGAaeaacqWFXoqyaaaabaGaem4uam1aaSbaaWqaaiab=f7aHbqabaWccqGHiiIZcqGFsc=ucqGGOaakcaqF4bGaeiykaKcabeqdcqGHris5aaWcbaGaemiCaa3aaSbaaWqaaiabdMgaPjabdQgaQbqabaaakiaawIJ=amaayaaabaWaaabuaeaacqWGqbaucqGGOaakcqWGtbWudaWgaaWcbaGae8NSdigabeaakiabcMcaPiab=r7aKnaaDaaaleaacqWGPbqAcqWGQbGAaeaacqWFYoGyaaGccqGGDbqxaSqaaiabdofatnaaBaaameaacqWFYoGyaeqaaSGaeyicI4Sae4NKWpLaeiikaGIaa0hEaiabcMcaPaqab0GaeyyeIuoaaSqaaiabdchaWnaaBaaameaacqWGPbqAcqWGQbGAaeqaaaGccaGL44paaeaacaaMc8UaaGPaVlaaykW7caaMc8UaaGPaVlaaykW7caaMc8UaaGPaVlaaykW7caaMc8UaaGPaVlaaykW7caaMc8UaaGPaVlaaykW7caaMc8UaaGPaVlaaykW7caaMc8Uaeyypa0ZaaSaaaeaacqaIXaqmaeaacqaIYaGmaaWaaabuaeaacqGGBbWwcqWGWbaCdaWgaaWcbaGaemyAaKMaemOAaOgabeaakiabgUcaRiabdchaWnaaBaaaleaacqWGPbqAcqWGQbGAaeqaaOGaey4kaSIaeGOmaiJaemiCaa3aaSbaaSqaaiabdMgaPjabdQgaQbqabaGccqWGWbaCdaWgaaWcbaGaemyAaKMaemOAaOgabeaaaeaacqWGPbqAcqGH8aapcqWGQbGAaeqaniabggHiLdGccqGGDbqxaeaacaaMc8UaaGPaVlaaykW7caaMc8UaaGPaVlaaykW7caaMc8UaaGPaVlaaykW7caaMc8UaaGPaVlaaykW7caaMc8UaaGPaVlaaykW7caaMc8UaaGPaVlaaykW7caaMc8Uaeyypa0ZaaabuaeaacqGGOaakcqWGWbaCdaWgaaWcbaGaemyAaKMaemOAaOgabeaakiabgkHiTiabdchaWnaaDaaaleaacqWGPbqAcqWGQbGAaeaacqaIYaGmaaGccqGGPaqkcqGGUaGlaSqaaiabdMgaPjabgYda8iabdQgaQbqab0GaeyyeIuoaaaaa@FAB4@

Thus normalising by length, the average base-pair distance is given by

D(x):=∑i<j(pij−pij2)L.
 MathType@MTEF@5@5@+=feaafiart1ev1aaatCvAUfKttLearuWrP9MDH5MBPbIqV92AaeXatLxBI9gBaebbnrfifHhDYfgasaacH8akY=wiFfYdH8Gipec8Eeeu0xXdbba9frFj0=OqFfea0dXdd9vqai=hGuQ8kuc9pgc9s8qqaq=dirpe0xb9q8qiLsFr0=vr0=vr0dc8meaabaqaciaacaGaaeqabaqabeGadaaakeaacqWGebarcqGGOaaktCvAUfeBSjuyZL2yd9gzLbvyNv2CaeHbwvMCKfMBHbaceeGaa8hEaGabaiaa+LcacaGF6aGaa4xpamaalaaabaWaaabeaeaacqGGOaakcqWGWbaCdaWgaaWcbaGaemyAaKMaemOAaOgabeaakiabgkHiTiabdchaWnaaDaaaleaacqWGPbqAcqWGQbGAaeaacqaIYaGmaaGccqGGPaqkaSqaaiabdMgaPjabgYda8iabdQgaQbqab0GaeyyeIuoaaOqaaiabdYeambaacqGGUaGlaaa@5044@

The last measure that we consider in this study is the valley index (*VI*) [21]. It can be regarded as an approximation to *D *(see below), and is meant to measure the number of "valleys" in the RNA folding landscape of **x**.

Formally it is defined as follows: List the suboptimal structures of **x **according to their free energies so that *S*_*opt*_, an MFE structure for **x**, is first and *S*_1_,..., *S*_*n *_are the next *n *structures on **x **with *E*_*opt *_≤ *E*_1 _≤ ⋯ ≤ *E*_*n*_. Put **S**_*subopt *_= {*S*_*opt*_, *S*_1_,..., *S*_*n*_}, and define

VI(x):=∑Sα,Sβ∈SsuboptdBPnorm(Sα,Sβ)w(α)w(β)∑Sα,Sβ∈Ssuboptw(α)w(β),
 MathType@MTEF@5@5@+=feaafiart1ev1aaatCvAUfKttLearuWrP9MDH5MBPbIqV92AaeXatLxBI9gBaebbnrfifHhDYfgasaacH8akY=wiFfYdH8Gipec8Eeeu0xXdbba9frFj0=OqFfea0dXdd9vqai=hGuQ8kuc9pgc9s8qqaq=dirpe0xb9q8qiLsFr0=vr0=vr0dc8meaabaqaciaacaGaaeqabaqabeGadaaakeaacqWGwbGvcqWGjbqscqGGOaaktCvAUfeBSjuyZL2yd9gzLbvyNv2CaeHbwvMCKfMBHbaceeGaa8hEaGabaiaa+LcacaGF6aGaa4xpamaalaaabaWaaabeaeaacqWGKbazdaWgaaWcbaGaemOqaiKaemiuaaLaemOBa4Maem4Ba8MaemOCaiNaemyBa0gabeaakiabcIcaOiabdofatnaaBaaaleaaiiGacqqFXoqyaeqaaOGaeiilaWIaem4uam1aaSbaaSqaaiab9j7aIbqabaGccqGGPaqkcqWG3bWDcqGGOaakcqqFXoqycqGGPaqkcqWG3bWDcqGGOaakcqqFYoGycqGGPaqkaSqaaiabdofatnaaBaaameaacqqFXoqyaeqaaSGaeiilaWIaem4uam1aaSbaaWqaaiab9j7aIbqabaWccqGHiiIZcaWFtbWaaSbaaWqaaiabdohaZjabdwha1jabdkgaIjabd+gaVjabdchaWjabdsha0bqabaaaleqaniabggHiLdaakeaadaaeqaqaaiabdEha3jabcIcaOiab9f7aHjabcMcaPiabdEha3jabcIcaOiab9j7aIjabcMcaPaWcbaGaem4uam1aaSbaaWqaaiab9f7aHbqabaWccqGGSaalcqWGtbWudaWgaaadbaGae0NSdigabeaaliabgIGiolaa=nfadaWgaaadbaGaem4CamNaemyDauNaemOyaiMaem4Ba8MaemiCaaNaemiDaqhabeaaaSqab0GaeyyeIuoaaaGccqGGSaalaaa@8B0A@

where w(α)=e−(Eα−Eopt)/RT
 MathType@MTEF@5@5@+=feaafiart1ev1aaatCvAUfKttLearuWrP9MDH5MBPbIqV92AaeXatLxBI9gBaebbnrfifHhDYfgasaacH8akY=wiFfYdH8Gipec8Eeeu0xXdbba9frFj0=OqFfea0dXdd9vqai=hGuQ8kuc9pgc9s8qqaq=dirpe0xb9q8qiLsFr0=vr0=vr0dc8meaabaqaciaacaGaaeqabaqabeGadaaakeaacqWG3bWDcqGGOaakiiGacqWFXoqycqGGPaqkcqGH9aqpcqWGLbqzdaahaaWcbeqaaiabgkHiTiabcIcaOiabdweafnaaBaaameaacqWFXoqyaeqaaSGaeyOeI0Iaemyrau0aaSbaaWqaaiabd+gaVjabdchaWjabdsha0bqabaWccqGGPaqkcqGGVaWlcqWGsbGucqWGubavaaaaaa@4342@ is the Boltzmann factor, and

dBPnorm(Sα,Sβ):=dBP(Sα,Sβ)L.
 MathType@MTEF@5@5@+=feaafiart1ev1aaatCvAUfKttLearuWrP9MDH5MBPbIqV92AaeXatLxBI9gBaebbnrfifHhDYfgasaacH8akY=wiFfYdH8Gipec8Eeeu0xXdbba9frFj0=OqFfea0dXdd9vqai=hGuQ8kuc9pgc9s8qqaq=dirpe0xb9q8qiLsFr0=vr0=vr0dc8meaabaqaciaacaGaaeqabaqabeGadaaakeaacqWGKbazdaWgaaWcbaGaemOqaiKaemiuaaLaemOBa4Maem4Ba8MaemOCaiNaemyBa0gabeaakiabcIcaOiabdofatnaaBaaaleaaiiGacqWFXoqyaeqaaOGaeiilaWIaem4uam1aaSbaaSqaaiab=j7aIbqabaGccqGGPaqkcqGG6aGocqGH9aqpdaWcaaqaaiabdsgaKnaaBaaaleaacqWGcbGqcqWGqbauaeqaaOGaeiikaGIaem4uam1aaSbaaSqaaiab=f7aHbqabaGccqGGSaalcqWGtbWudaWgaaWcbaGae8NSdigabeaakiabcMcaPaqaaiabdYeambaacqGGUaGlaaa@4F09@

Note that our definition of *VI *differs slightly from the Kitagawa *et al*.'s definition since we use normalised base-pair distance, *d*_*BPnorm*_, rather than the coarse-grained tree metric in their study. The suboptimal structures *S*_1_,..., *S*_*n *_are randomly sampled with probabilities equal to their Boltzmann weights using the program RNAsubopt [22]. We sample 300 structures resulting in between 16 (regulatory) and 300 (telomerase) unique structures.

In principle, the valley index for an RNA with a low number of valleys in the folding landscape should be low, whereas an RNA with a multi-valley folding landscape should have a correspondingly higher index. Note that the sums in the definition on *VI *are taken over all structures in a set of suboptimal structures within a certain energy distance from the MFE. If the energy distance is increased this set of structures will eventually include all the sequences in the ensemble S(x)
 MathType@MTEF@5@5@+=feaafiart1ev1aaatCvAUfKttLearuWrP9MDH5MBPbIqV92AaeXatLxBI9gBaebbnrfifHhDYfgasaacH8akY=wiFfYdH8Gipec8Eeeu0xXdbba9frFj0=OqFfea0dXdd9vqai=hGuQ8kuc9pgc9s8qqaq=dirpe0xb9q8qiLsFr0=vr0=vr0dc8meaabaqaciaacaGaaeqabaqabeGadaaakeaatCvAUfeBSjuyZL2yd9gzLbvyNv2CaeHbbjxAHXgaiqaacaWFtbGaeiikaGsegyvzYrwyUfgaiuqacaGF4bGaeiykaKcaaa@3CC3@. In this situation, in view of the definition of *w*(*α*) it follows that the valley index of **x **can be rewritten as

VI(x)=∑Sα,Sβ∈S(x)dBPnorm(Sα,Sβ)(e−(Eα−Eopt)/RT)(e−(Eβ−Eopt)/RT)∑Sα,Sβ∈S(x)(e−(Eα−Eopt)/RT))(e−(Eβ−Eopt)/RT)=∑Sα,Sβ∈S(x)dBPnorm(Sα,Sβ)(e−Eα/RT)(e−Eβ/RT)∑Sα,Sβ∈S(x)(e−Eα/RT)∑Sβ∈S(x)(e−Eβ/RT)=∑Sα,Sβ∈S(x)dBPnorm(sα,Sβ)(e−Eα/RT)(e−Eβ/RT)Z2=∑Sα,Sβ∈S(x)∑P(Sα)P(Sβ)dBPnorm(Sα,Sβ)=2D(x).
 MathType@MTEF@5@5@+=feaafiart1ev1aaatCvAUfKttLearuWrP9MDH5MBPbIqV92AaeXatLxBI9gBamrtHrhAL1wy0L2yHvtyaeHbnfgDOvwBHrxAJfwnaebbnrfifHhDYfgasaacH8akY=wiFfYdH8Gipec8Eeeu0xXdbba9frFj0=OqFfea0dXdd9vqai=hGuQ8kuc9pgc9s8qqaq=dirpe0xb9q8qiLsFr0=vr0=vr0dc8meaabaqaciaacaGaaeqabaWaaeGaeaaakqaaeeqaaiabdAfawjabdMeajjabcIcaOmXvP5wqSXMqHnxAJn0BKvguHDwzZbqehyvzYrwyUfgaiuqacaWF4bGaeiykaKIaeyypa0ZaaSaaaeaadaaeqaqaaiabdsgaKnaaBaaaleaacqWGcbGqcqWGqbaucqWGUbGBcqWGVbWBcqWGYbGCcqWGTbqBaeqaaOGaeiikaGIaem4uam1aaSbaaSqaaGGaciab+f7aHbqabaGccqGGSaalcqWGtbWudaWgaaWcbaGae4NSdigabeaakiabcMcaPiabcIcaOiabdwgaLnaaCaaaleqabaGaeyOeI0IaeiikaGIaemyrau0aaSbaaWqaaiab+f7aHbqabaWccqGHsislcqWGfbqrdaWgaaadbaGaem4Ba8MaemiCaaNaemiDaqhabeaaliabcMcaPiabc+caViabdkfasjabdsfaubaakiabcMcaPiabcIcaOiabdwgaLnaaCaaaleqabaGaeyOeI0IaeiikaGIaemyrau0aaSbaaWqaaiab+j7aIbqabaWccqGHsislcqWGfbqrdaWgaaadbaGaem4Ba8MaemiCaaNaemiDaqhabeaaliabcMcaPiabc+caViabdkfasjabdsfaubaakiabcMcaPaWcbaGaem4uam1aaSbaaWqaaiab+f7aHbqabaWccqGGSaalcqWGtbWudaWgaaadbaGae4NSdigabeaaliabgIGioprtHrhAL1wy0L2yHDdarGqtHrhAL1wy0L2yHDdaiyaacqqFsc=ucqGGOaakcaWF4bGaeiykaKcabeqdcqGHris5aaGcbaWaaabeaeaacqGGOaakcqWGLbqzdaahaaWcbeqaaiabgkHiTiabcIcaOiabdweafnaaBaaameaacqGFXoqyaeqaaSGaeyOeI0Iaemyrau0aaSbaaWqaaiabd+gaVjabdchaWjabdsha0bqabaWccqGGPaqkcqGGVaWlcqWGsbGucqWGubavcqGGPaqkaaGccqGGPaqkcqGGOaakcqWGLbqzdaahaaWcbeqaaiabgkHiTiabcIcaOiabdweafnaaBaaameaacqGFYoGyaeqaaSGaeyOeI0Iaemyrau0aaSbaaWqaaiabd+gaVjabdchaWjabdsha0bqabaWccqGGPaqkcqGGVaWlcqWGsbGucqWGubavaaGccqGGPaqkaSqaaiabdofatnaaBaaameaacqGFXoqyaeqaaSGaeiilaWIaem4uam1aaSbaaWqaaiab+j7aIbqabaWccqGHiiIZcqqFsc=ucqGGOaakcaWF4bGaeiykaKcabeqdcqGHris5aaaaaOqaaiabg2da9maalaaabaWaaabeaeaacqWGKbazdaWgaaWcbaGaemOqaiKaemiuaaLaemOBa4Maem4Ba8MaemOCaiNaemyBa0gabeaakiabcIcaOiabdofatnaaBaaaleaacqGFXoqyaeqaaOGaeiilaWIaem4uam1aaSbaaSqaaiab+j7aIbqabaGccqGGPaqkcqGGOaakcqWGLbqzdaahaaWcbeqaaiabgkHiTiabdweafnaaBaaameaacqGFXoqyaeqaaSGaei4la8IaemOuaiLaemivaqfaaOGaeiykaKIaeiikaGIaemyzau2aaWbaaSqabeaacqGHsislcqWGfbqrdaWgaaadbaGae4NSdigabeaaliabc+caViabdkfasjabdsfaubaakiabcMcaPaWcbaGaem4uam1aaSbaaWqaaiab+f7aHbqabaWccqGGSaalcqWGtbWudaWgaaadbaGae4NSdigabeaaliabgIGiolab9jj8tjabcIcaOiaa=HhacqGGPaqkaeqaniabggHiLdaakeaadaaeqaqaaaWcbaGaem4uam1aaSbaaWqaaiab+f7aHbqabaWccqGGSaalcqWGtbWudaWgaaadbaGae4NSdigabeaaliabgIGiolab9jj8tjabcIcaOiaa=HhacqGGPaqkaeqaniabggHiLdGccqGGOaakcqWGLbqzdaahaaWcbeqaaiabgkHiTiabdweafnaaBaaameaacqGFXoqyaeqaaSGaei4la8IaemOuaiLaemivaqfaaOGaeiykaKYaaabeaeaacqGGOaakcqWGLbqzdaahaaWcbeqaaiabgkHiTiabdweafnaaBaaameaacqGFYoGyaeqaaSGaei4la8IaemOuaiLaemivaqfaaOGaeiykaKcaleaacqWGtbWudaWgaaadbaGae4NSdigabeaaliabgIGiolab9jj8tjabcIcaOiaa=HhacqGGPaqkaeqaniabggHiLdaaaaGcbaGaeyypa0ZaaSaaaeaadaaeqaqaaiabdsgaKnaaBaaaleaacqWGcbGqcqWGqbaucqWGUbGBcqWGVbWBcqWGYbGCcqWGTbqBaeqaaOGaeiikaGIaem4Cam3aaSbaaSqaaiab+f7aHbqabaGccqGGSaalcqWGtbWudaWgaaWcbaGae4NSdigabeaakiabcMcaPiabcIcaOiabdwgaLnaaCaaaleqabaGaeyOeI0Iaemyrau0aaSbaaWqaaiab+f7aHbqabaWccqGGVaWlcqWGsbGucqWGubavaaGccqGGPaqkcqGGOaakcqWGLbqzdaahaaWcbeqaaiabgkHiTiabdweafnaaBaaameaacqGFYoGyaeqaaSGaei4la8IaemOuaiLaemivaqfaaOGaeiykaKcaleaacqWGtbWudaWgaaadbaGae4xSdegabeaaliabcYcaSiabdofatnaaBaaameaacqGFYoGyaeqaaSGaeyicI4Sae0NKWpLaeiikaGIaa8hEaiabcMcaPaqab0GaeyyeIuoaaOqaaiabdQfaAnaaCaaaleqabaGaeGOmaidaaaaaaOqaaiabg2da9maaqababaWaaabuaeaacqWGqbaucqGGOaakcqWGtbWudaWgaaWcbaGae4xSdegabeaakiabcMcaPiabdcfaqjabcIcaOiabdofatnaaBaaaleaacqGFYoGyaeqaaOGaeiykaKIaemizaq2aaSbaaSqaaiabdkeacjabdcfaqjabd6gaUjabd+gaVjabdkhaYjabd2gaTbqabaaabaaabeqdcqGHris5aOGaeiikaGIaem4uam1aaSbaaSqaaiab+f7aHbqabaGccqGGSaalcqWGtbWudaWgaaWcbaGae4NSdigabeaakiabcMcaPiabg2da9iabikdaYiabdseaejabcIcaOiaa=HhacqGGPaqkaSqaaiabdofatnaaBaaameaacqGFXoqyaeqaaSGaeiilaWIaem4uam1aaSbaaWqaaiab+j7aIbqabaWccqGHiiIZcqqFsc=ucqGGOaakcaWF4bGaeiykaKcabeqdcqGHris5aOGaeiOla4caaaa@A28E@

Thus, 12VI(x)
 MathType@MTEF@5@5@+=feaafiart1ev1aaatCvAUfKttLearuWrP9MDH5MBPbIqV92AaeXatLxBI9gBaebbnrfifHhDYfgasaacH8akY=wiFfYdH8Gipec8Eeeu0xXdbba9frFj0=OqFfea0dXdd9vqai=hGuQ8kuc9pgc9s8qqaq=dirpe0xb9q8qiLsFr0=vr0=vr0dc8meaabaqaciaacaGaaeqabaqabeGadaaakeaadaWcaaqaaiabigdaXaqaaiabikdaYaaacqWGwbGvcqWGjbqscqGGOaaktCvAUfeBSjuyZL2yd9gzLbvyNv2CaeHbwvMCKfMBHbaceeGaa8hEaiabcMcaPaaa@3E25@ can be thought of as an approximation of *D*(*x*) in case the set *S*_*subopt *_(**x**) used in the computation of *VI*(**x**) is a proper subset of S(x)
 MathType@MTEF@5@5@+=feaafiart1ev1aaatCvAUfKttLearuWrP9MDH5MBPbIqV92AaeXatLxBI9gBaebbnrfifHhDYfgasaacH8akY=wiFfYdH8Gipec8Eeeu0xXdbba9frFj0=OqFfea0dXdd9vqai=hGuQ8kuc9pgc9s8qqaq=dirpe0xb9q8qiLsFr0=vr0=vr0dc8meaabaqaciaacaGaaeqabaqabeGadaaakeaatCvAUfeBSjuyZL2yd9gzLbvyNv2CaeHbbjxAHXgaiqaacaWFtbGaeiikaGsegyvzYrwyUfgaiuqacaGF4bGaeiykaKcaaa@3CC3@.

## Results and discussion

### Comparison of measures

**Figure 1:**
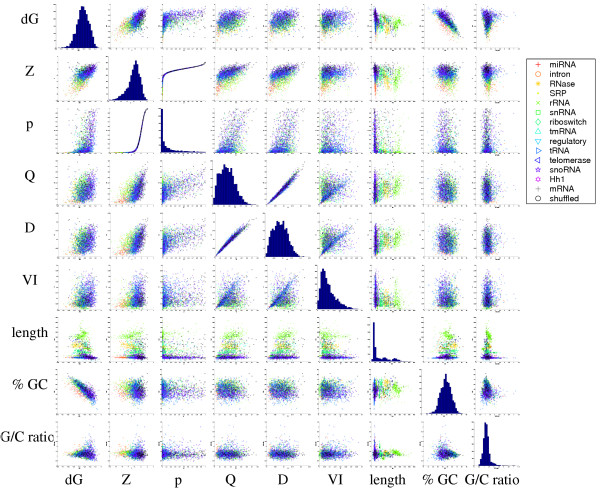
**Correlations between measures**. Correlations between all the different measures for all the data sets are shown. The diagonal figures show the distributions of the measures.

The six measures that we investigated are correlated to varying degrees; see Table 2 and Figure 1. The measures *Q *and *D *are highly correlated (correlation coefficient = 0.98), which could be due to the fact that they are both computed using McCaskill base pair probabilities, *p*_*ij*_. Also, as expected, the *Z*-score and *p*-value are strongly correlated, but not in a linear fashion (see Figure 1). We see that the *Z*-scores are more sensitive for low values than the *p*-values (e.g. all *Z*-scores below -3 correspond to a *p*-value of 0.0), and so *Z*-scores are more informative.

**Table 2:** Correlations between measures. Correlation coefficients between the different measures, values above 0.5 are in bold.

*dG*	**1.00**								
*Z*	**0.62**	**1.00**							
*p*	0.48	**0.74**	**1.00**						
*Q*	0.33	**0.52**	**0.51**	**1.00**					
*D*	0.31	**0.51**	0.48	**0.98**	**1.00**				
*VI*	0.19	0.23	0.19	0.29	0.33	**1.00**			
length	-0.26	-0.19	-0.10	0.32	0.28	-0.17	**1.00**		
%GC	**-0.78**	-0.13	-0.12	-0.08	-0.06	-0.05	0.18	**1.00**	
G/C ratio	0.03	-0.05	-0.02	0.01	0.01	0.06	0.00	-0.14	**1.00**
	*dG*	*Z*	*p*	*Q*	*D*	*VI*	length	%GC	G/C ratio

The statistic *dG *is weakly correlated to all other measures. However, it is interesting to note that *dG *is negatively correlated to %-GC. This is to be expected since GC base pairs have lower energy than the other possible base-pairings. The miRNA family is an exception to this rule, since it has low *dG *values, but an average %-GC of about 50%, see Figure 1.

Table 2 shows that the correlation between *VI *and the other measures is low over all families. However, Figure 1 indicates that for a subset of all the sequences the correlation between *VI *and *Q *or *D *is very strong. This is also confirmed by computing the correlation coefficients for the 15 RNA families separately. miRNA, SRP, tRNA, telomerase, and Hh1 show strong correlations (> 0.65) between *VI *and *Q *or D, whereas the corresponding correlations for rRNA, snRNA, riboswitch, regulatory, and snoRNA are weak (< 0.3).

### Comparison between RNA families

In general, we deem an RNA sequence to have a stable secondary structure if the measures *dG*, *Z*, and *p *are significantly lower than the corresponding values for the shuffled control data sets. To check whether this was the case for the different data sets, we applied a Mann-Whitney rank sum test [23]. This test compares two data sets and computes the probability that the two data sets are sampled from the same distribution. Unlike the *t*-test, the Mann-Whitney test is distribution free since it compares the ranks of the data values instead of the data values themselves.

**Figure 2:**
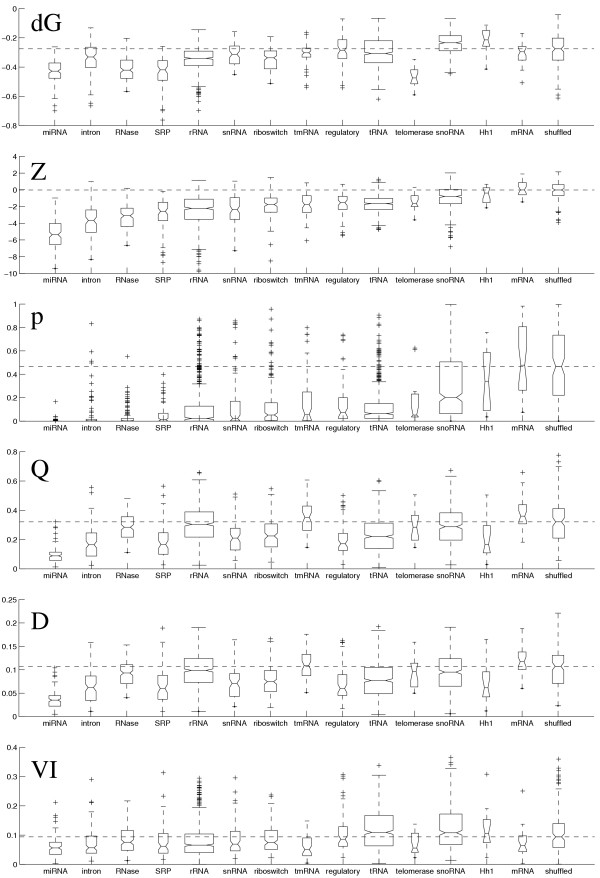
**Box and whisker plots of *dG*, *Z*, *p*, *Q*, *D*, and *VI***. Box and whisker plots displaying medians, quartiles and range of the measures *dG*, *Z*, *p*, *Q*, *D*, and *VI*. The lines of the box are at the lower quartile, median, and upper quartile values. The box width is proportional to the number of sequences in the data set. The whisker lines extend from each end of the box to the most extreme data value or have a maximal length of 1.5 times the box height. Data points beyond the ends of the whiskers are marked by +.

At a significance level of 99% the Mann-Whitney test indicated that *Z *and *p *are higher for the shuffled data set than for any of the real RNA data sets, except for mRNA and Hh1. The same held for the normalised energy *dG*, except for the tmRNA, tRNA, regulatory and snoRNA families. This result agrees with those observed in [10], that ncRNAs have significantly lower *Z*-score than unstructured sequences. This can also be seen in Figure 2.

The measures *Q *and *D *can be used to indicate whether a sequence folds into a unique secondary structure or into several alternative structures [24]. The riboswitch data set consists of sequences known to have alternative structures, and so we expected the values of *Q *and *D *to be rather high for this data set. We did find this to be the case, but surprisingly they were also as high or even higher for other data sets (see Figure 2).

The high values of *Q *and *D *obtained for the mRNA and shuffled data sets is probably due to the fact that these RNAs are unstructured, and hence there are many alternative possible structures. This could also explain the values of *Q *and *D *for tmRNA, since tmRNAs are to a large extent mRNA-like (large parts of such molecules are unstructured). Other RNA families like tRNA and RNAse have tertiary interactions that aren't included in secondary structure, which explains their relatively high *Q- *and *D*-values. The interaction of rRNAs and snoRNAs with proteins and other RNAs most likely stabilise their native structures, even though alternative structures are possible.

**Figure 3:**
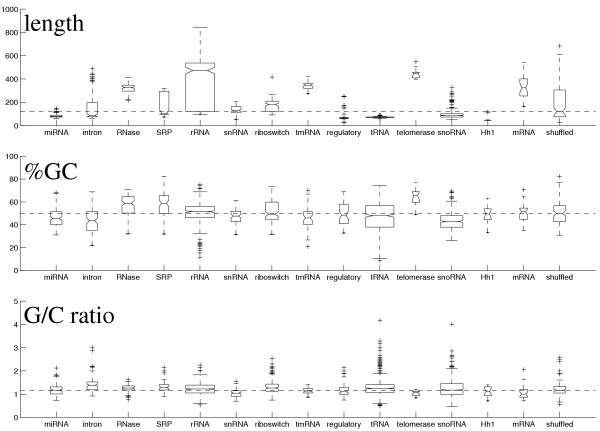
**Box and whisker plots of length, %GC, and G/C ratio**. Box and whisker plots displaying medians, quartiles and range of the sequence length, %GC, and G/C ratio for all our test data sets. The lines of the box are at the lower quartile, median, and upper quartile values. The box width is proportional to the number of sequences in the data set. The whisker lines extend from each end of the box to the most extreme data value or have a maximal length of 1.5 times the box height. Data points beyond the ends of the whiskers are marked by +.

The values of our measures for the telomerase sequences were unexpected. Telomerase has low energy per base, yet it has a rather high *Z*-score compared to the other ncRNAs. The high stability of this molecule is most likely due to an unusual sequence composition; the telomerase sequences have a high %-GC level, 65% (see Figure 3). The high values of *Q *and *D *suggest that the telomerase sequences have alternative structures.

The miRNAs have very stable structures, indicated by low *Z *and *dG*, especially in view of their %GC level (~50%). This has previously been observed in [11]. The miRNAs also have low values of *Q, D*, and *VI*, indicating a unique structure.

### Comparison with previous studies

Seffens and Digby [6] examined 51 mRNA sequences and observed that they have lower folding energy than shuffled versions of the sequences preserving mono- but not dinucleotide frequencies. Shortly after, Workman and Krogh examined 46 of the 51 mRNAs and showed that they do not have lower folding energy than shuffled versions of the sequences, when the dinucleotide frequencies are preserved [8]. In our study, in which sequences were shuffled so as to preserve both mono- and dinucleotide frequencies, we confirm that mRNAs do not have lower folding energy than shuffled sequences. In [8] a small sample of rRNA and tRNA sequences were also investigated and it was indicated that rRNA, but not tRNA has lower folding energy than dinucleotide shuffled sequences. Our study, with significantly more data, agrees with their findings for rRNA, but differs for tRNAs, which we found to have significantly lower *Z*-scores than shuffled sequences. Rivas and Eddy [9] argue that secondary structure alone is generally not significant for the detection of ncRNA, but note that ncRNAs have slightly lower folding energies than shuffled sequences. Note that in [9] sequences are shuffled preserving mononucleotides only, whereas in our study we shuffled sequences preserving dinucleotide frequencies. Rivas and Eddy computed *Z*-scores for a large set of tRNAs, and even though we adopt a different shuffling procedure, our results for tRNA are in good agreement with Rivas and Eddy's findings.

Kitagawa *et al. *[21] observed that five snRNAs have low folding energies compared to shuffled sequences. Our studies confirm this observation, and in general we found that snRNA sequences have lower folding energies than shuffled sequences with the same dinucleotide frequency. Kitagawa *et al. *also computed *VI *values for the same five snRNAs, and observed that the values varied considerably (indicating that some have uni-valley landscapes while other have multi-valley landscapes). Although we used a variant of *VI*, we also found that the *VI *value varies considerably for different snRNA sequences.

Bonnet *et al. *observed that miRNAs have considerably lower folding energy than dinucleotide shuffled sequences, unlike tRNA and rRNA [11]. Our studies confirm this observation, although Bonnet *et al. *investigated shorter regions of the rRNA, while we investigated full rRNA sequences.

In our study, we found the mean *Z*-scores (and *p*-values) to be significantly lower for ncRNAs (except the Hammerhead type I family) than for the shuffled sequences (although the *Z*-scores for mRNA were not lower). This is in agreement with recent results presented in [10], where it is shown that non-coding RNAs have lower *Z*-scores than coding RNAs for a selection of RNA families (tRNA, Hammerhead type III, a regulatory element (SECIS), SRP, snRNA (U1 and U2), mRNA (divided into coding sequence and 5'- and 3'-untranslated regions)).

## Conclusion

We have studied six previously defined measures for predicting how well an RNA molecule is expected to fold (*dG*, *Z*, *p*, *Q*, *D*, and *VI*), and applied them to a large collection of RNAs from the Rfam database. We found all of these measures to be correlated to some degree. The measures *Z *and *p *are strongly correlated, but *Z *is more sensitive than *p*. Since *dG *is a measure of MFE it is strongly correlated to the nucleotide composition of the sequence, and so a low *dG *does not necessarily imply a stable structure. Hence, it is probably sufficient to use *Z *as opposed to *p *and *dG*. For the families that we used in this study, we found the mean *Z*-scores (and *p*-values) to be significantly lower for ncRNAs than for the shuffled sequences.

The three measures *Q*, *D *and *VI *can be regarded as measures of the ruggedness of the RNA folding landscape. Both *Q *and *D *are computed from the partition function and are thus strongly correlated, and so either of them is probably sufficient for measuring ruggedness. The valley index *VI *can be viewed as an approximation of the average base-pair distance *D *(see Methods section), and so there is no advantage in computing *VI*, especially since it is slow to compute, whereas *D *can be computed efficiently. RNA families having high values of *D *(and *Q*) were either unstructured RNA sequences, long RNA sequences that fold with the help of proteins, or RNAs with alternative folds or pseudoknot structures.

Thus, in summary, we expect that rather than using all of *dG*, *Z*, *p*, *Q*, *D*, and *VI *to predict how well an RNA molecule folds, that it is sufficient to use only *Z *and *D *(or *Q*). Our studies suggest that a combination of *Z*-score and *D *value might be useful for identifying well-defined RNA structures, such as the miRNAs (in agreement with results presented in [11]), and, based on our results, we expect that variations of these measures (such as the alignment *Z*-scores described in [12]), will provide a useful tool for the general problem of RNA structure identification.

## Authors' contributions

EF was involved in selecting the data sets from Rfam and implementing the analyses. PPG developed the ideas presented in the paper and was involved in selecting the data sets from Rfam and implementing the analyses. VM was involved in developing the ideas presented in the paper. All authors contributed to the writing of this manuscript. All authors read and approved the final manuscript.

## Supplementary Material

Additional File 1 **Data sets**. This zip-file contains all the sequences we have used for this study. Click here for file
